# Cellular Automata-Based Application for Driver Assistance in Indoor Parking Areas †

**DOI:** 10.3390/s16111921

**Published:** 2016-11-15

**Authors:** Cándido Caballero-Gil, Pino Caballero-Gil, Jezabel Molina-Gil

**Affiliations:** Department of Computer Engineering and Systems, University of La Laguna, La Laguna 38271, Tenerife, Spain; pcaballe@ull.es (P.C-G.); jmmolina@ull.es (J.M.-G.)

**Keywords:** indoor parking, intelligent transport system, smartphone application, cellular automata, location-based service

## Abstract

This work proposes an adaptive recommendation mechanism for smart parking that takes advantage of the popularity of smartphones and the rise of the Internet of Things. The proposal includes a centralized system to forecast available indoor parking spaces, and a low-cost mobile application to obtain data of actual and predicted parking occupancy. The described scheme uses data from both sources bidirectionally so that the centralized forecast system is fed with data obtained with the distributed system based on smartphones, and vice versa. The mobile application uses different wireless technologies to provide the forecast system with actual parking data and receive from the system useful recommendations about where to park. Thus, the proposal can be used by any driver to easily find available parking spaces in indoor facilities. The client software developed for smartphones is a lightweight Android application that supplies precise indoor positioning systems based on Quick Response codes or Near Field Communication tags, and semi-precise indoor positioning systems based on Bluetooth Low Energy beacons. The performance of the proposed approach has been evaluated by conducting computer simulations and real experimentation with a preliminary implementation. The results have shown the strengths of the proposal in the reduction of the time and energy costs to find available parking spaces.

## 1. Introduction

The growing number of vehicles on the road in recent years has resulted in a general lack of parking availability. This situation implies many disadvantages when commuting, such as driver discomfort and time consumption, which cause additional stress to drivers, and thus, increase the risk of road accidents when looking for available parking spaces. Furthermore, this issue is an important cause of extra carbon dioxide emissions, which deteriorate the environment, especially when many people are simultaneously searching for available parking spaces at peak hours. Therefore, innovative systems that meet the high demands of efficient parking management are urgently needed. This paper proposes the use of wireless technologies of smartphones to develop a smart parking management system that can improve the usage of parking facilities and the user experience of drivers by simplifying the process of finding available parking.

In the design of a solution based on smartphones and wireless technologies, an additional difficulty appears when vehicles enter indoor installations, such as indoor parking facilities where the use of GPS (Global Positioning System) or indoor triangulation is not possible or precise enough to provide useful data. In some indoor parking facilities, a simple system is used that automatically detects the arrival and departure of vehicles through various sensors, in order to conclude whether there is still any available parking space, as well as to display global updated information on parking space occupancy. However, since this occupancy information is given to all drivers at the same time, it could produce new congestion because many drivers would drive to the same zones. The system here proposed offers each driver with a specific recommended route to minimize the expected search time. In particular, the proposal estimates the occupancy from entrance counters and the information sensed by the smartphones running the application, this information is centrally collected, and then the server running the forecast method disseminates the processed information to the vehicles with the application, which then calculates the recommended route.

In this work, a novel approach for a parking solution based on a combination of available advanced technologies and a mathematical forecasting method is introduced [1]. On the one hand, it involves a smartphone application that uses accurate and cheap technologies for indoor positioning. On the other hand, cellular automata are used to model the usual behaviour of drivers in parking facilities. In particular, the system applies the idea behind a modified version of the game of life to capture some features of parking occupancy based on common user behaviour, in order to try to reduce the time to find available parking spaces.

The described service has been defined to be used from the beginning so that it can be gradually adopted and smartly improved through its use in order to raise the success rate. The proposal has been simulated and the obtained conclusion is that the service is low-cost and energy-efficient for end users, and an effective solution for managers of parking facilities.

This paper is structured as follows. Section 2 briefly describes some related work. Section 3 presents the main features of the proposed scheme including a parking locator service that uses smartphones, a forecast parking system based on cellular automata, and a driver-assistance method. Some results for a basic simulation and a functional demonstration of the service are shown in Section 4. Finally, this work ends with some conclusions and open problems.

## 2. Related Work

This work proposes a novel parking service that combines a smartphone application to indicate parking occupancy through different wireless technologies together with a system to forecast availability in parking spaces. The following sections briefly revise some works related to each of those two issues.

### 2.1. Detection of Indoor Parking Occupancy

Existing intelligent parking services could be improved by taking advantage of a new generation of technological devices and infrastructures [2,3]. On the one hand, WLANs (Wireless Local Area Networks) and 2G/3G or LTE (Long Term Evolution) networks enable ubiquitous Internet access with a smartphone. For instance, the work [4] fuses WLAN and sensors of smartphones to develop an application for intelligent parking services. On the other hand, the concept of a connected car is being substantiated by major vehicle and tech manufacturers, which are developing related products and services, such as Android Auto by Google (San Francisco, CA, USA) or Apple CarPlay (Cupertino, CA, USA) [5,6,7,8,9].

Indoor mapping is another critical tool necessary to develop a new generation of intelligent parking systems for indoor installations [10]. Several companies, such as Aisle411 (St. Louis, MO, USA), Google, Micello (Santa Clara, CA, USA), Navteq (Chicago, IL, USA), Nokia (Uusimaa, Finland) or Point Inside (Bellevue, WA, USA), have developed various indoor map products for a range of points of interest, such as shopping centres and airports, throughout North America, Europe and Japan.

The usefulness of proposals for optimizing indoor parking heavily relies on the precision of indoor localization [11]. In particular, intelligent parking systems require an accurate indoor positioning system and the acquisition of parking space occupancy status information in real time. This type of information can be obtained by various detection sensors [12], which can be divided, depending on their position, into two categories: intrusive and non-intrusive sensors [13]. On the one hand, intrusive sensors have to be installed underneath or across the pavement in every parking space, thus involving a high cost. For instance, the work [14] utilizes a type of sensors for vehicle detection that has to be embedded into the ground. The proposal [15] also uses underground sensors combined with overhead sensors. On the other hand, the main advantage of non-intrusive sensors is that they can be easily installed in existing facilities without requiring any major additional hardware or installation cost because they only require fixing on the ceiling or on the sides of the parking spaces. However, some of them could present many problems in some parking topologies. Two examples of non-intrusive systems, based, respectively, on RFID (Radio Frequency IDentification) and minimally-intrusive sensors, are [16,17]. Although the installation and maintenance of non-intrusive sensors do not result in invasive procedures or traffic disruptions, and are cheaper than intrusive sensors, their cost is higher than the cost of the proposal here described, which is based on QR (Quick Response) codes, NFC (Near Field Communication) tags and BLE (Bluetooth Low Energy) beacons. This is not the first work that has addressed the parking problem by using QR [18,19] or NFC [20]. Thus, we know that the main problem in using these technologies is that they require the driver to be close in order to be able to use them. This is the reason why the proposed system offers, as an easier option, the use of BLE technology. The BLE-based solution here proposed for indoor parking tries to take advantage of its low energy consumption and the possibility to place beacons where WLAN access points cannot be placed. In particular, the proposal presented here has been implemented with BLE devices within the Physical Web [21], a new project from Google’s Chrome team that provides lightweight discovery and an interaction layer to the IoT (Internet of Things). This system is cheap and does not require a beacon per parking space. Instead, just one beacon is necessary per section including from eight to 16 rows of cars, depending on the walls and other obstacles.

### 2.2. Parking Forecast

**Figure 1:**
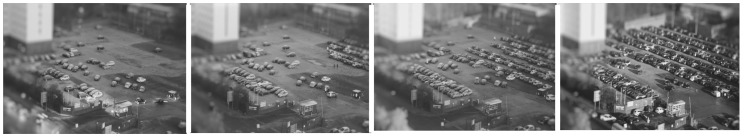
Photos of the progress of actual parking occupancy.

Figure 1 shows, with an example, a typical behaviour of drivers in a parking space. As can be seen, users park their vehicles according to a pattern, for instance in the spaces closest to the exits.

In general, the behaviour of vehicles in parking spaces may be modelled like the population growth in urban environments, which has been studied in several works like [22,23,24,25]. Markov chain models and queuing theory are used in the work [26] based on the forecasting method of demand for parking described in [27], which estimates possible availability of parking spaces by considering the historical data. Other researchers have used fuzzy logic-based decision-making to combine parking availability, future demand and user requirements [28,29,30]. The forecast of parking availability in real time has also been studied through intelligent approaches like [31], which uses discrete models to simulate the demand, so that, based on its probability of availability, it allocates the demand. In order to provide real-time information on the availability of parking, the work [32] introduces a smart parking system that uses historical parking data in a prediction model, and in order to learn historical models of parking availability per street, the paper [33] presents a back-end based approach. The work [34] also uses collected data on parking availability to forecast available parking spaces by applying a wavelet neural network. Finally, the paper [35] describes an approach to forecast modelling the occupancy rate, not only of indoor areas, but also of on-street parking places, taking into account current and future events that influence the general demand for parking spaces.

**Figure 2:**
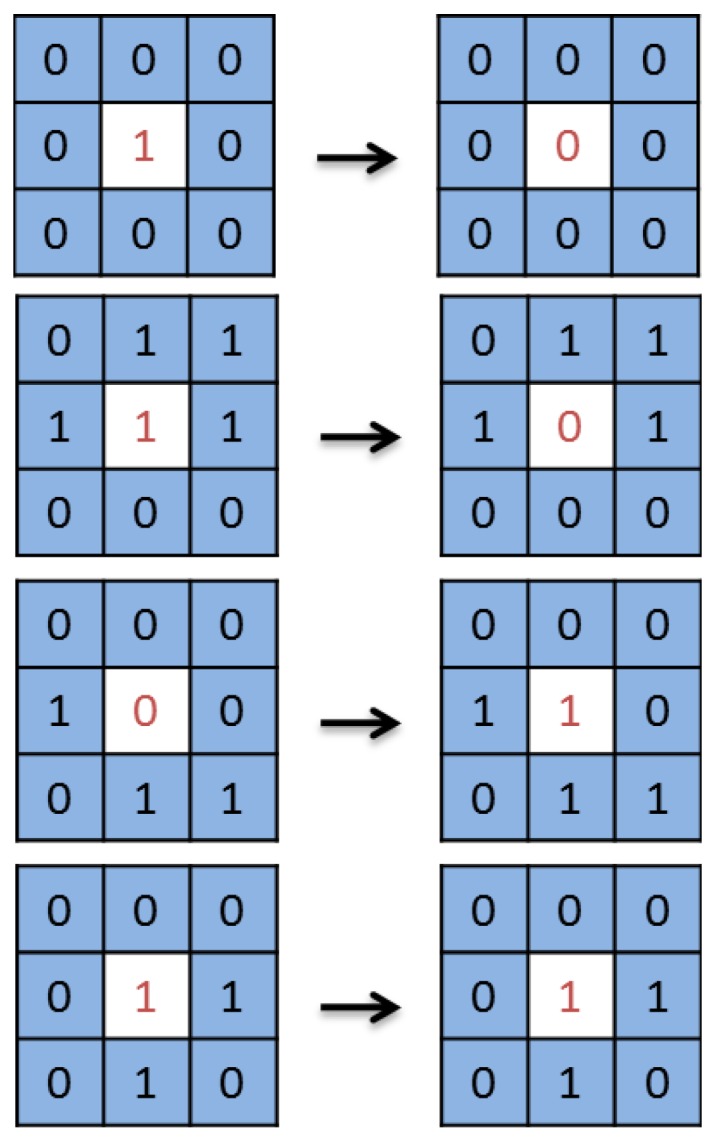
Game of life.

The system to forecast parking availability defined here is completely different from the aforementioned because it is based on a version of the game of life, which is a cellular automaton introduced by John Conway in 1970 [36]. According to that model, cells are self-replicating machines [37] that reproduce themselves autonomously. In the game of life, every cell has two possible states (dead or alive), depending on its neighbourhood formed by the eight surrounding cells. Its name is because every cell symbolizes a living organism that needs its neighbours to survive. Figure 2 reflects the following four rules that define the automaton. Cells with fewer than two living neighbours die of loneliness. Cells with more than three living neighbours die of overcrowding. Dead cells with three living neighbours become alive. Living cells with two or three living neighbours stay alive.

To the best of our knowledge, only the recent works [38,39,40] present systems for parking recommendation based on CA (Cellular Automata).

The main differences between this paper and the works [38,39] are that those systems have a focus not directed towards indoor facilities, use different technologies, and are not completed with a practical application for the drivers. In particular, the work [38] reports on the development of an agent-based cruising-for-parking simulation using CA, while the research [39] uses the direction of travel along with the request for parking the vehicle, in order to reduce congestion and avoid unnecessary traffic manoeuvres.

With respect to the work [40], there are many differences with the proposal here defined. Firstly, such a paper is based on the information sent from the on-board devices on the cars via a WLAN because it assumes that the parking facility provides WLAN access to the cars. In addition, it defines different parking zones, assigns probabilities to find available spaces in each area, uses a graph to represent the parking facility, and assumes that drivers park at any available parking space they find. Finally, what is more important, it states that a cellular automaton is used, but it is not specified, and its use is for modelling traffic. In contrast, the scheme here defined assumes neither WLAN access nor choice of the first available parking, and does not use zones, probabilities or graphs. This work is based on a new defined cellular automaton that is used for modelling parking user behaviour.

## 3. Proposed Scheme

### 3.1. Smartphone-Based System for Parking Occupancy Detection

**Figure 3:**
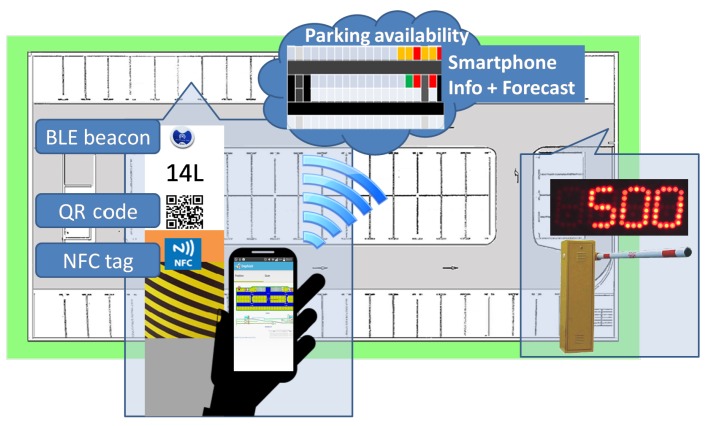
Low-cost indoor parking system.

This paper proposes a low-cost solution for indoor parking facilities, which requires no fixed physical component to control parking availability. The idea, as shown in Figure 3, consists of two main components. The first component is a smartphone application that sends data on actual occupancy to a server and receives from it information to help drivers find parking spaces that are available with a high probability. The second component is a server that manages the data on the actual occupancy of parking spaces received from the smartphone application and the entrance counter, applies a predictive method on them, and sends back to the smartphone a recommendation about where to park. The first component is described in this section, while the second one is described in the next section.

**Figure 4:**
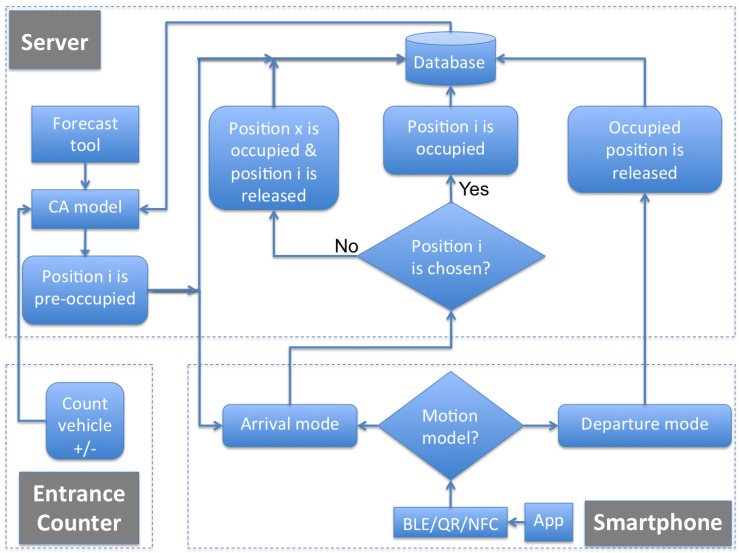
High-level architecture of the indoor parking system.

As shown in Figure 4, the proposed platform has been implemented through a distributed architecture of smartphones and a centralized server.

On the one hand, the mobile application uses a combination of technologies formed by QR codes, NFC tags and BLE Physical Web Beacons to determine actual occupancy, and sends back these data to a server. Depending on the technologies (BLE, NFC, QR) available on the smartphone, the proposed system uses and integrates different types of data. If it has Bluetooth, the smartphone application first uses the Android platform SDK (Software Development Kit) to do a scanning of the presence of any BLE signal nearby at the parking facility. If it detects any, the application does measurements of the received RSSI (Received Signal Strength Indicator) from all detected beacons, and sends these measurements to the server together with the MAC (Media Access Control) address received from each scanned beacon. With these data, at the server, it is possible to compute the approximate position of the vehicle depending on the locations of the detected BLE devices. Regarding the use of BLE for indoor positioning, it has been proved that adopting BLE beacons as signal sources and RSSI as measurement is a reasonable solution because this technology allows obtaining over 95% of correct estimation rate [41]. Otherwise, if the smartphone has no Bluetooth, the driver must use either NFC or QR to scan the signal or code located at every parking space by approximating the smartphone to the NFC device or printed QR code available near each parking space. In both cases, the scanned data are sent through the application to the server so that the location of the vehicle can be stored. The application is able to automatically identify whether the vehicle is occupying or releasing a parking space. Again, if the smartphone has Bluetooth, the hands-free device of the vehicle allows automatic knowledge of when the vehicle is being started or when it is being stopped, concluding that the corresponding parking space is being released or occupied. Otherwise, if the smartphone has no Bluetooth, the driver must use either NFC or QR to scan the signal or code located at every parking space to indicate to the server when it is releasing a parking space.

On the other hand, the server centralizes all the information regarding available, occupied and possibly occupied parking spots and shows data regarding occupied and recently released parking spaces. Taking into account that not all drivers will have the application running on their smartphones, the predictive tool described in the next section is used to estimate which parking spaces are available in the parking facility. Thanks to the entrance counter, the server knows when each vehicle arrives at the parking facility. Thus, through the forecast tool, it chooses for that vehicle a possibly available position i and marks it as pre-occupied. If the vehicle is not using the smartphone application, the position i chosen for it remains as possibly occupied in the database. Otherwise, if the vehicle is using the smartphone application but occupies another position x, the position x changes to occupied and the position i is released in the database. When a vehicle leaves the parking facility, also thanks to the entrance counter, the server knows it. In this case, a position is always released in the database.

Service providers establish the interfaces to bridge parking facilities and users over the Internet, and develop tools to create and maintain the parking facilities database. Operators of parking facilities use information from user applications and the predictive tool to monitor the occupancy of each parking space and to publish the parking space occupancy online forecasting information in real time. Thus, the Internet is used in the proposed scheme to connect the different parties, including parking facilities, customers and service providers.

The information on each parking space includes its geographical coordinates, occupancy status, etc. A lightweight client program on each user smartphone allows accessing the service through the Internet to collect information about the parking facility and to store and send data about the current parking space occupancy.

The server and the database have been developed using several frameworks that allow the creation of efficient and scalable solutions. In particular, the involved technologies are the following:

- Node.js is an open source, cross-platform runtime environment for server-side and networking applications, which are written in JavaScript, and can be run within the Node.js runtime on multiple platforms. It is commonly used for real-time web applications.
- Express.js is a Node.js web application framework, designed for building single-page, multi-page, and hybrid web applications.
- MongoDB is a cross-platform document-oriented database. Classified as an NoSQL (Not only SQL) database, MongoDB avoids the traditional table-based relational database structure in favour of JSON (JavaScript Object Notation) like documents with dynamic schemas.
- Mongoose is a framework that allows the modelling of data from a MongoDB database, allowing CRUD (Create, Read, Update and Delete) operations.
- Angular.js is an open-source web application framework for client-side MVC (Model-View-Controller) architecture.
- Bootstrap is a front-end that works as an interface for the user, unlike the server-side code that resides on the back-end or server.
- Embedded JavaScript cleans the HTML out of JavaScript with client side templates.

The parking service exploits an accurate positioning solution based on QR codes, NFC tags and BLE Physical Web Beacons. In its development, the user operations are conducted on the parking client software. The application has been developed for the Android operating system and has been tested with a Samsung smartphone (Samsung, Suwon, Korea) that runs Android 4.4 (Google, Mountain View, CA, USA). The software development has been done with Android Studio. The main APIs (Application Programming Interfaces) and resources for the smartphone application are:

- An image-based floor map, used as indoor map.
- Physical Web API, used to acquire the measurements of the physical web beacons for indoor positioning.
- ZXing API, used to capture QR codes [42].

**Figure 5:**
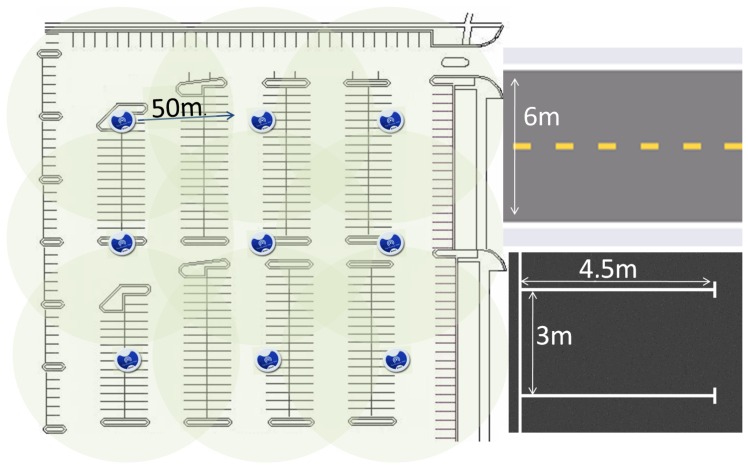
Example of parking facility with nine Bluetooth Low Energy (BLE) beacons.

BLE beacons have been used for indoor positioning because this kind of positioning solution is cost-efficient and can be operated in conjunction with communication services. In addition, the interaction between BLE beacons and the smartphone can be automated so that the user does not have to do anything anything either on arrival or on departure. The inconvenience is that location is not as precise as with QR or NFC solutions. Unlike traditional solutions, which are usually based on specific hardware tags for positioning, the proposed solution uses the built-in hardware of a smartphone to collect the signals of the beacons, and performs positioning estimation using information from the server. Experiments with BLE-based solutions for indoor positioning have shown that the number of required beacons is lower compared with solutions based on other technologies. Thus, for instance, the work [43] proved that in a room of 204 m${}^{2}$ partially separated by a wall in the middle, five beacon nodes produced a fine accuracy confidence, and the main problem was that often at least one beacon is hidden behind the wall. Parking areas do not have many dividing walls so this problem does not highly affect the proposed scheme. We performed our own experiments and checked that in the parking of Figure 5, nine beacons were enough to cover all parking spaces with very high precision.

QR codes and NFC tags are cheap technologies that can be used to solve the indoor location problem. These technologies are really convenient for the proposal because they can easily be used to remind users where a vehicle is parked, which can be used as an added value of the application to encourage its use. In this case, to find the best walking route, the pathfinding framework [44] has been used in the implementation. These technologies are easy to use but have the inconvenience that they are not automatic. Thus, in order to occupy a parking space in the server, the user has to take a picture of the QR code or put their smartphone close to the NFC tag. Thus, it is possible that many users will not use the technology because they will forget to perform this simple step. The release of a parking space is automatic when the smartphone detects the connection with the hands-free system.

### 3.2. CA-Based System for Parking Forecast

The described mobile application allows identifying parking spaces by using either BLE or QR/NFC. However, when such data are missing because not all vehicles are provided with hands-free devices and/or drivers may forget to scan codes or tags when occupying or releasing spaces, the CA-based system presented in this section is essential for the operation of the proposal.

In order to model the occupancy behaviour of parking spaces, different use cases have been studied. For instance, in a parking facility with just one Point Of Exit (POE), most users try to park their vehicles in places close to the POE. Indeed, there are some studies that state a relationship between the choice of parking space and the distance to ticket machines, entrances, exits, final destinations, etc. [45]. In addition, distinct behaviour can be distinguished inside the parking facility depending on the hour of the day, the day of the week, etc. Thus, occupancy of parking spaces seems not to be a random decision but to follow clear rules. In this work, a CA-based model is used to forecast parking occupancy.

The CA used in our model to describe driver behaviour in indoor parking is defined according to three basic stages that can be distinguished during parking occupancy: Stage 1 (Filling), Stage 2 (Swapping) and Stage 3 (Emptying). The first and last stages correspond, respectively, to the opening and closing of the parking facilities. Vehicle behaviour with respect to a parking space is defined in each stage depending on the space state (available or occupied). In this work, a modified Conway’s game of life is used for the parking model [36], where living cells correspond to occupied parking spaces while dead cells are available parking spaces. The forecast tool based on this automaton is more accurate during Stage 1 (Filling) than in the other two stages. In that stage, if the tool fails to recommend an available parking space, it is highly probable that there are other available parking spaces near the recommended one.

The used model defines a matrix where different positions are parking spaces or lanes. Roads can be horizontal or vertical and can have one or two lanes, and every parking space must be accessible from a road. The algorithm includes a loop whose iterations define the state of each parking space.

**Figure 6:**
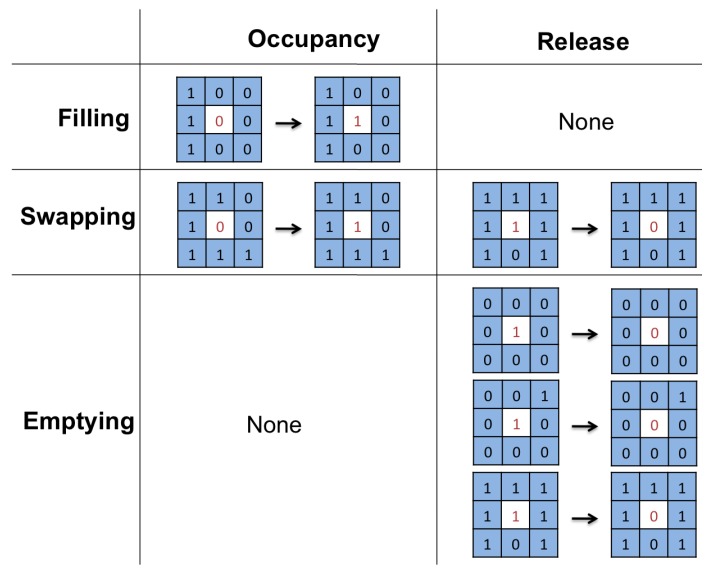
Rules defining occupancy and release for each stage.

Figure 6 shows an example of the rules applied in the proposed system, defined following the current behaviour pattern seen in parking spaces in order to simulate it. Thus, the system calculates the next value of each cell x(i,j) taking into account the values of all its neighbours x(i,j−1), x(i,j+1), x(i−1,j), x(i+1,j), x(i−1,j−1), x(i+1,j−1), x(i−1,j+1) and x(i+1,j+1).

This model follows from a study of indoor facilities with parking fees, where the conclusion is that driver behaviour can be modelled using a modified version of the game of life. During the Filling Stage, vehicles tend to occupy parking spaces in a predefined way. During the Emptying Stage, vehicles tend to release parking spaces in a random way. Finally, during the Swapping Stage, available parking spaces mainly surrounded by occupied ones are quickly occupied, available parking spaces mainly surrounded by available ones remain available, occupied parking spaces mainly surrounded by available ones are quickly released, and occupied parking spaces mainly surrounded by occupied ones are quickly released.

The adjustment of the game of life to the proposed model is shown below:

- Filling Stage
  - Empty cells with three or more neighbours become occupied.
  - Empty cells with two or fewer neighbours remain empty.
  - Occupied cells remain occupied.
- Swapping Stage
  - Empty cells with six or more neighbours become occupied.
  - Empty cells with five or fewer neighbours remain empty.
  - Occupied cells with six or more neighbours are released.
  - Occupied cells with three to five neighbours remain occupied.
  - Occupied cells with two or fewer neighbours are released.
- Emptying Stage
  - Empty cells remain empty.
  - Occupied cells with seven or more neighbours are released.
  - Occupied cells with three to six neighbours become occupied.
  - Occupied cells with two or fewer neighbours are released.

The interpretation of the above rules is the following. The first three rules of the Filling stage describe a strategy according to which vehicles tend to park near popular places like POEs. The three rules of the Swapping stage can be summed up as behaviour similar to the game of life. Finally, the three rules of the Emptying stage try to reflect the usual behaviour in parking facilities when users are leaving because the parking facility is about to close. This cellular automaton is deterministic and the stationary state depends only on the initial conditions of actual occupancy data provided by the smartphones of drivers. This automaton tries to mimic the complex interactions between vehicles looking for available parking spaces. However, the model does not fully reflect the behaviour of all drivers because not all of them react in the same way, as some vary their parking behaviour without any obvious reasons. Therefore, during the practical implementation of the system under real conditions during a period of time, it could be convenient to introduce some type of random noise in the model so that it would reflect a more realistic description including this type of user.

### 3.3. Driver-Assistance System

A simple adaptive recommendation mechanism for smart parking is included in the proposal.

The application computes the fastest route to the pre-occupied position recommended by the forecast tool, on the basis of the parking map information provided by the parking management. Afterwards, it shows the route to the driver, including navigation directions to the destination like turn left, turn right, or go straight ahead.

When reaching the recommended position, if the vehicle cannot park there, the application reinitiates automatically to the arrival mode so that a new recommended closer position and faster route are provided.

If positions close to each other were recommended to many users at the same time, the routes to these positions would get congested. Thus, to avoid this, the server tool computes several best positions according to the forecast model and the actual position of each vehicle, and presents different recommendations chosen at random to those users arriving together.

In this work, the goal is not only to reduce time and fuel consumption but also to allow the easy finding of a parking space in indoor parking areas. Thus, the innovative CA mechanism combined with the adaptive recommendation mechanism provides the following benefit. The driver can park in a preferred parking space by avoiding probably occupied parking spaces, so the lack of delay during the search will prevent them from feeling annoyed.

## 4. Simulation and Implementation

The aforementioned CA-based model has been used to simulate with the Matlab program (MathWorks, Natick, MA, USA) the behaviour of drivers in indoor parking facilities. On the one hand, the input to this simulation is provided by the data sent through the proposed smartphone application from vehicles in the parking facility. On the other hand, the output of the simulation is used to predict availability and occupancy of parking spaces. Due to the existence of general limitations of simulations used for prediction, it is worthwhile to mention that any model is only as good as its assumptions. Thus, the choice of assumptions for the CA-based simulation is considered essential in this proposal. In order to simulate the behaviour of vehicles and parking occupancy in indoor parking facilities, we have used the location of the exits as initial seed for the CA-based model, assuming that these points are the key to the behaviour pattern.

**Figure 7:**
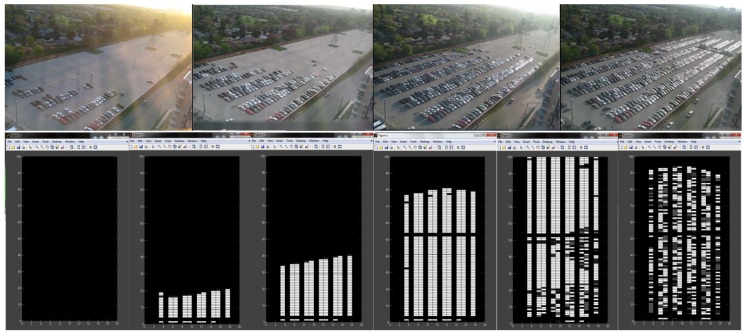
Simulation of cellular automata (**bottom**) to forecast the progress of parking occupancy (**top**) using Matlab.

The actual situation seen in the first images of Figure 7 has been recreated using Matlab, as shown at the bottom of the image of Figure 7, where each cell corresponds to a parking space. With this simulation of the proposed CA-based model, available parking spaces can be forecasted, especially during the Filling stage.

Thus, Figure 7 shows a simulation of driver behaviour in the scenario of a parking facility with one POE, once an adequate CA initial state has been chosen that takes into account where the POE is. As can be seen, the simulation grows in a similar way to the real scenario shown in Figure 7.

**Figure 8:**
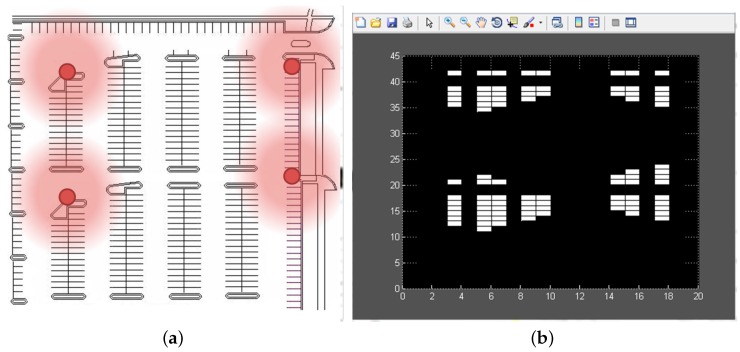
(**b**) Simulation of (**a**) parking facility with four Point Of Exit (POEs).

The proposed tool allows modelling different parking spaces with different topologies, including parking facilities with more or fewer rows or columns of parking spaces, POEs, lanes, sections, etc. For instance, the map on the left in Figure 8 shows a parking facility with four POEs, and, as can be seen, once an adequate CA initial state has been chosen taking into account where the POEs are, the model reflects that users tend to park near the four access points.

**Figure 9:**
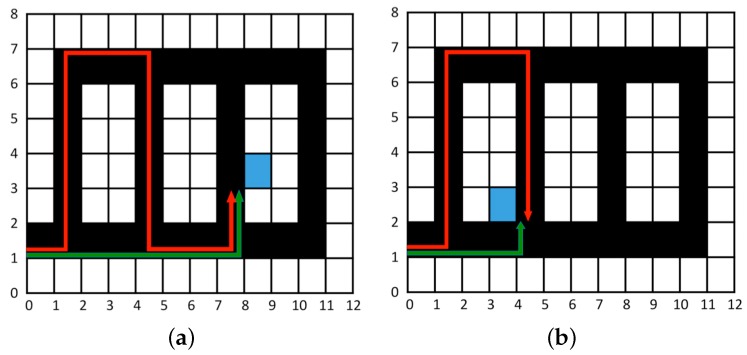
Efficient (green) and inefficient (red) routes to find an available parking space. (**a**) in (8,3); (**b**) in (4,2).

Regarding the influence of knowledge on the optimization of the route to find an available parking space, Figure 9 shows through a simple example the difference between an efficient and an inefficient route from the parking entrance to an available space. If we identify each parking space by the coordinates of the one of its four vertices that is accessible and closer to the parking entrance, the available space indicated in Figure 9a is identified by the coordinates (8,3). In this example, it is assumed that the inefficient route consists in going wandering inside the parking facility, crossing completely each section till finding an available space. Thus, according to this model, there are some parking spaces, such as those identified by the coordinates (2,1), (3,1), (5,7), (6,7), (8,1) and (9,1), which cannot be visited through the inefficient route. The measurements to define in general the efficiency in terms of traversed distance in both routes, $D^{ef}$ and $D^{inef}$ from the entrance located at position $\left(i_{0},j_{0}\right)$ to an available parking space located at position (i,j) in the parking facility organized into columns shown in Figure 9, are given by the following expressions:

(1) $$D^{ef}\left(\left(i_{0},j_{0}\right),\left(i,j\right)\right)=|i-i_{0}|\cdot S_{i}+\left|j-j_{0}\right|\cdot S_{j},$$

(2) $$D^{inef}\left(\left(i_{0},j_{0}\right),\left(i,j\right)\right)=\left\{\begin{array}{cc} D^{ef}\left(\left(i_{0},j_{0}\right),\left(i,j\right)\right)+(\lceil \frac{|i-i_{0}|}{d}\rceil -1)\cdot S_{sec} & \;if\;i\equiv 1\;or\;2\;or\;3\;(mod\;2d)\\ D^{ef}\left(\left(i_{0},j_{0}\right),\left(i,j\right)\right)+\left\lceil \frac{|i-i_{0}|}{d}\right\rceil \cdot S_{sec}-\left|j-j_{0}\right|\cdot 2S_{j} & \;if\;i\equiv 4\;or\;5\;(mod\;2d), \end{array}\right.$$

where

- $S_{i}$: Size of each row.
- $S_{j}$: Size of each column.
- $S_{sec}$: Size of each section by columns.
- *d*: Distance between the neighbouring columns.

As aforementioned, these expressions are obtained assuming that the driver in the efficient case goes directly from the entrance to the closest available parking space located at (i,j), while in the inefficient case, he/she completely traverses each section till he/she reaches the available parking space located at (i,j). In the example of Figure 9, $S_{i}=S_{j}=1$, $S_{sec}=6$, d=3 and $\left(i_{0},j_{0}\right)=\left(0,1\right)$. There we can see that the difference between the efficient and the inefficient routes depends on whether the number of full sections traversed by the inefficient route is even or odd. Thus, on the one hand, for the instance of the space (8,3) shown in Figure 9a, $D^{inef}\left((0,1),(8,3)\right)=D^{ef}\left((0,1),(8,3)\right)+\left(\lceil 8/3\rceil -1\right)\times 6$, so the difference between both routes is 12, which corresponds exactly with the distance for traversing two full sections. On the other hand, for the instance of the space (4,2) shown in Figure 9b, $D^{inef}\left((0,1),(4,2)\right)=D^{ef}\left((0,1),(4,2)\right)+\left\lceil 4/3\right\rceil \times 6-\left(2-1\right)\times 2$, so the difference between both routes is 12−2=10, which corresponds to the distance of traversing less than two sections.

This simple example shows a typical behaviour pattern because, in general, users try to park as close as they can to a POE. Thus, if they do not know where the best available parking space is, they search through all the parking facilities. Otherwise, if users know where a possibly available space is, they go directly to it, saving time and fuel and increasing their comfort.

**Figure 10:**
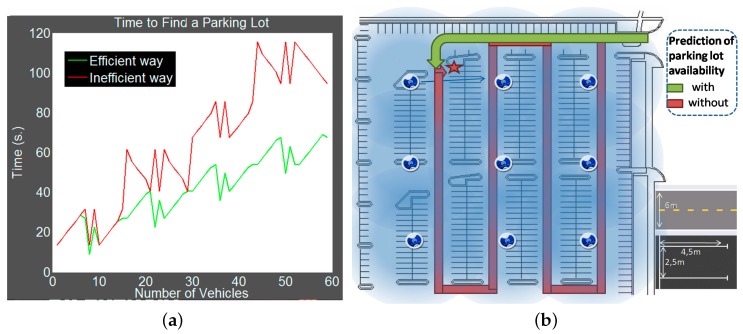
Example of times to find an available parking space. (**a**) chart; (**b**) simulated parking.

Thus, in the case of the parking facility to the right of Figure 10, the image on the left shows the difference between the distances that a user has to traverse to find an available parking space depending on the information he/she knows. The method indicated as an efficient way corresponds to drivers who use the proposed application, while the time corresponding to the rest of the vehicles there is indicated with the inefficient way tag. This figure also shows the relationship between the number of vehicles and the search time, reflecting that, in both cases, the larger the number of vehicles, the longer the search time. As can be seen, users who have additional information go directly, while users without such information go wandering inside the parking facility until they find a preferred available parking space. Consequently, since, in this second case, a high number of vehicles could provoke a traffic jam inside the facility, the more vehicles there are in the parking facility, the greater the difference is between the times to park in both cases. These results demonstrate the improvement provided by the proposal, even in a very simple parking scenario like that one.

**Figure 11:**
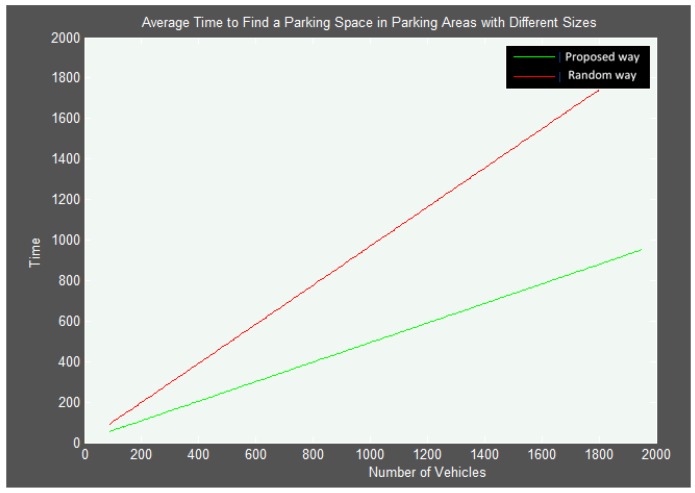
Average time to find an available parking space.

A wider analysis has been done through simulations using the CA-based algorithm and a beta mobile application in indoor smart parking environments, where Matlab was used with more than 100 simulations in several scenarios in parking facilities ranging from 50 to 2000 parking spaces and from one to 10 POEs. Figure 11 shows the average time to find an available parking space considering a number of vehicles between 0 and 2000, and all the cases from 0 to 100% occupancy. As can be seen, the growth of the average time with respect to the number of vehicles, represented by the slope of the straight line in Figure 11, is lower for the proposal than in the absence of an optimized scheme. In addition, it can be seen that the searching time increases as the number of parking spaces increases due to the fact that more congestion is provoked near the POEs. This clearly indicates the advantage of using the proposal especially in large parking facilities with a high flow of vehicles, which is just where more parking problems appear.

**Figure 12:**
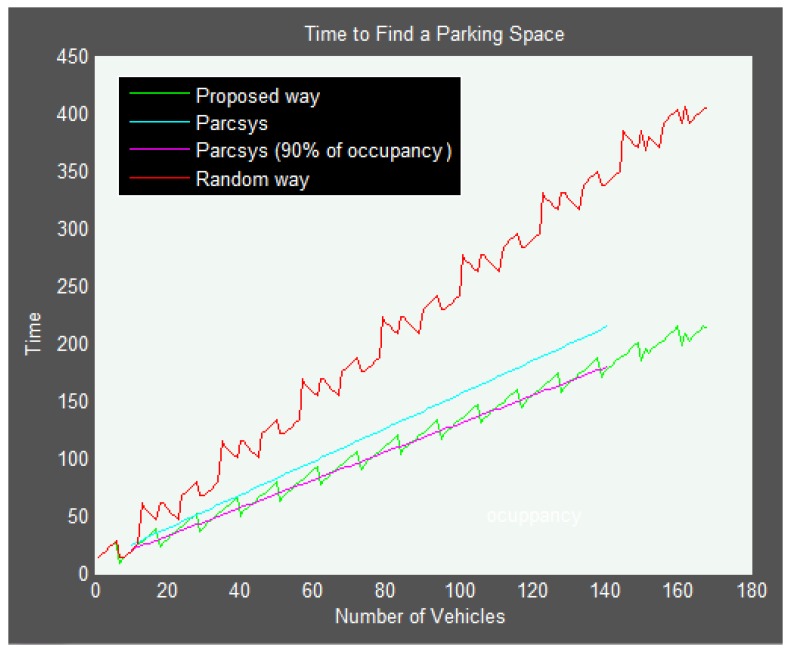
Comparative analysis.

The above results have been used for a comparative study between this work and a prior proposal. In particular, the scheme chosen for the comparison is PARC (Parking Access and Revenue Control), a system that helps to reduce searching times in parking facilities and whose methods can be found in [46]. Figure 12 shows average searching times to find an available parking space using different schemes. In order to compare the time to find an available parking space, the used parking model is the same as the one used in the paper [46], where a parking facility with 152 parking spaces is considered, with parking spaces on the right, on the left and in two rows in the middle. This distribution has been implemented in Matlab to calculate the performance time of the proposed system.

It can be observed that the average performance of the system proposed here is equal to that of the PARC system with 90% occupancy, and better than that of the PARC system on average. The reason why the proposed system is better than the PARC system when the rate of occupancy is lower than 90% could be due to the fact that PARC uses sensors at key locations to divide the parking area into smaller zones in order to calculate the parking occupancy in such zones, which results in a loss of information. Furthermore, in contrast with the PARC system, with the proposed system, it is not necessary to install either VMS (Variable Message Sign) panels or sensors in the parking facility.

The performed evaluation has been carried out over parking facilities with just one floor, but many parking facilities have several levels. In order to handle such multi-storey parking facilities, the proposed method could be modified by considering that, in the same way that happens with the distances from the POEs, parking levels are not equally favoured by users due to the difference of distances from the level/s of the entrance/s. Thus, the proposal here presented can be modified by replicating the CA-based model at each level depending on its POEs, and changing the driver-assistance system so that it computes the recommendations by assigning lower probabilities to those levels that are more distant from the level/s of the entrance/s.

The conclusion of the above analysis is that the proposed parking service based on both forecast and availability of information from users’ smartphones improves the user experience of customers through the parking recommendation and the reminder of the location of their parked vehicles. In addition, it is also demonstrated that the system is energy-saving because the route of each vehicle to find a preferred available parking space is shorter.

## 5. Conclusions

This work introduces an optimized mechanism for finding available indoor parking spaces using a CA-based mechanism and a mobile application that takes advantage of a combination of wireless technologies. The proposal is a complete service that helps the driver to find an available parking space more quickly, and offers an orientation service to take him/her to his/her parked vehicle. The proposed predictive tool is based on a modified version of the game of life to forecast the behaviour of vehicles inside the parking facility. The designed tool uses a mobile application based on QR codes, NFC tags and/or BLE beacons to provide the CA-based model with real data of parking occupancy. On the one hand, the server application offers an availability forecast based on estimated and actual occupancy of parking spaces. On the other hand, the mobile application helps the final user to find the recommended parking space quickly. From the results of experimentation, it has been found that the proposal achieves substantial improvements for smart parking in indoor installations in terms of reducing the time and traversed distance to find an available parking space, so the conclusion is that the proposal is more convenient than the conventional opportunism system and other previous methods. As part of work in progress, when the system is fully implemented in practice, true and false positives and negatives could be used as metrics to identify other influencing factors and possible random behaviour, and improve the model to forecast available spaces with higher precision. In addition, the system can also be enhanced with the addition of a tool to estimate the probability that an available parking space at a suggested location gets filled up before arrival, and to forecast future demand for parking spaces.

## References

[1] Caballero-Gil C., Molina-Gil J., Caballero-Gil P. (2015). Low-Cost Service to Predict and Manage Indoor Parking Spaces. *Ubiquitous Computing and Ambient Intelligence. Sensing, Processing, and Using Environmental Information*, Proceedings of the 9th International Conference on UCAmI 2015.

[2] Levine U., Shinar A., Shabtai E. (2011). System and Method for Parking Time Estimations. U.S. Patent.

[3] Vlahogianni E.I., Kepaptsoglou K., Tsetsos V., Karlaftis M.G. (2016). A real-time parking prediction system for smart cities. J. Intell. Transp. Syst..

[4] Liu J., Chen R., Chen Y., Pei L., Chen L. (2012). iParking: An intelligent indoor location-based smartphone parking service. Sensors.

[5] Google (2016). Android Auto. http://www.android.com/auto.

[6] Apple (2016). Car Play. http://www.apple.com/ios/carplay.

[7] BMW (2016). ConnectedDrive. http://www.bmw.es/connecteddrive.

[8] Ford (2016). Ford Sync. http://www.ford.com/technology/sync.

[9] Generals Motors (2016). On Star. https://www.onstar.com.

[10] Khoshelham K., Elberink S.O. (2012). Accuracy and resolution of kinect depth data for indoor mapping applications. Sensors.

[11] Liu H., Darabi H., Banerjee P., Liu J. (2007). Survey of wireless indoor positioning techniques and systems. IEEE Trans. Syst. Man Cybern. C (Appl. Rev.).

[12] Zheng Y., Rajasegarar S., Leckie C. Parking availability prediction for sensor-enabled car parks in smart cities. Proceedings of the IEEE Tenth International Conference on Intelligent Sensors, Sensor Networks and Information Processing.

[13] Mimbela L.E.Y., Klein L.A. (2003). Summary of Vehicle Detection and Surveillance Technologies Used in Intelligent Transportation Systems. http://www.fhwa.dot.gov/ohim/tvtw/vdstits.pdf.

[14] Prasad A.G., Sharath U., Amith B., Supritha B.R., Asokan S., Hegde G.M. Fiber Bragg Grating sensor instrumentation for parking space occupancy management. Proceedings of the International Conference on Optical Engineering.

[15] Haoui A., Kavaler R., Varaiya P. (2008). Wireless magnetic sensors for traffic surveillance. Transp. Res. C.

[16] Ganesan K., Vignesh K. Automated Parking Slot Allocation using RFID Technology. Proceedings of the International Symposium on Signal Processing and Its Applications.

[17] Vera-Gómez J.A., Quesada-Arencibia A., García C.R., Suárez-Moreno R., Guerra-Hernández F. (2016). An intelligent parking management system for urban areas. Sensors.

[18] Hammadi O.A., Hebsi A.A., Zemerly M.J., Ng J.W. Indoor localization and guidance using portable smartphones. Proceedings of the IEEE/WIC/ACM International Joint Conferences on Web Intelligence and Intelligent Agent Technology.

[19] Costa-Montenegro E., González-Castaño F., Conde-Lagoa D., Barragans-Martínez A., Rodríguez-Hernández P., Gil-Castiñeira F. QR-maps: An efficient tool for indoor user location based on qr-codes and google maps. Proceedings of the IEEE Consumer Communications and Networking Conference.

[20] Kim M.S., Lee D.H., Kim K.N.J. A study on the NFC-based mobile parking management system. Proceedings of the IEEE International Conference on Information Science and Applications.

[21] (2014). Physical Web. http://google.github.io/physical-web/.

[22] Clarke K., Hoppen S., Gaydos L. (1997). A self-modifying cellular automaton model of historical. Environ. Plan. B.

[23] Herold M., Goldstein N.C., Clarke K.C. (2003). The spatiotemporal form of urban growth: Measurement, analysis and modeling. Remote Sens. Environ..

[24] Martens K., Benenson I. (2008). Evaluating urban parking policies with agent-based model of driver parking behavior. Transp. Res. Rec..

[25] Caicedo F., Robuste F., Lopez-Pita A. (2006). Parking management and modeling of car park patron behavior in underground facilities. Transp. Res. Rec..

[26] Klappenecker A., Lee H., Welch J.L. (2014). Finding available parking spaces made easy. Ad Hoc Netw..

[27] Caliskan M., Barthels A., Scheuermann B., Mauve M. Predicting parking lot occupancy in vehicular ad-hoc networks. Proceedings of the IEEE Vehicular Technology Conference.

[28] Teodorovic D., Lucic P. (2006). Intelligent parking systems. Eur. J. Oper. Res..

[29] Chen Z., Xia J.C., Irawan B. (2013). Development of fuzzy logic forecast models for location-based parking finding services. Math. Probl. Eng..

[30] Leephakpreeda T. (2007). Car-parking guidance with fuzzy knowledge-based decision making. Build. Environ..

[31] Caicedo F., Blazquez C., Miranda P. (2012). Prediction of parking space availability in real time. Expert Syst. Appl..

[32] Fabusuyi T., Hampshire R.C., Hill V., Sasanuma K. A Predictive Model and Evaluation Framework for Smart Parking: The Case of ParkPGH. Proceedings of the 18th ITS World Congress.

[33] Richter F., Di Martino S., Mattfeld D.C. Temporal and spatial clustering for a parking prediction service. Proceedings of the IEEE International Conference on Tools with Artificial Intelligence.

[34] Ji Y., Tang D., Blythe P., Guo W., Wang W. (2015). Short-term forecasting of available parking space using wavelet neural network model. IET Intell. Transp. Syst..

[35] David A., Overkamp K., Scheuerer W. Event-oriented forecast of the occupancy rate of parking spaces as part of a parking information service. Proceedings of the 7th World Congress on Intelligent Systems.

[36] Gardner M. (1970). Mathematical games: The fantastic combinations of John Conways new solitaire game life. Sci. Am..

[37] Von Neumann J., Burks A.W. (1966). Theory of self-reproducing automata. IEEE Trans. Neural Netw..

[38] Horni A., Montini L., Waraich R.A., Axhausen K.W. (2013). An agent-based cellular automaton cruising-for-parking simulation. Transp. Lett..

[39] Horng G.J. (2014). Using cellular automata for parking recommendations in smart environments. PLoS ONE.

[40] Sun W., Shibata N., Kenmotsu M., Yasumoto K., Ito M. (2015). A method for navigating cars in multilevel parking facility. J. Inf. Process..

[41] Kajioka S., Mori T., Uchiya T., Takumi I., Matsuo H. Experiment of indoor position presumption based on RSSI of Bluetooth LE beacon. Proceedings of the IEEE Global Conference on Consumer Electronics.

[42] (2016). Official Zxing (“Zebra Crossing”) Project. https://github.com/zxing/zxing/.

[43] Dahlgren E., Mahmood H. (2014). Evaluation of Indoor Positioning Based on Bluetooth Smart Technology. Master’s Thesis.

[44] (2015). PathFinding Project. http://qiao.github.io/PathFinding.js/visual/.

[45] Van der Waerden P., Borgers A., Timmermans H. Travelers micro-behavior at parking lots: A model of parking choice behavior. Proceedings of the Annual Meeting of the Transportation Research Board.

[46] Caicedo F. (2009). The use of space availability information in PARC systems to reduce search times in parking facilities. Transp. Res. C Emerg. Technol..

